# Alloimmunisation fœto-maternelle Rhésus grave à propos d'un cas et revue de la littérature

**DOI:** 10.11604/pamj.2015.22.137.3508

**Published:** 2015-10-14

**Authors:** Benkerroum Zineb, Lachiri Boutaina, Lazrak Ikram, Moussaoui Rahali Driss, Dehayni Mohammed

**Affiliations:** 1 Service de Gynécologie Obstétrique, Hôpital Militaire d'instruction Mohammed V, Avenue des Far Hay Riad, Rabat, Maroc

**Keywords:** Alloimmunisation, anasarque fœtal, pic systolique, transfusion in utéro, prophylaxie, Alloimmunisation, fetal hydrops, systolique peak, in utero transfusion, prophylaxy

## Abstract

L'incompatibilité fœto-maternelle Rhésus peut être à l'origine d'un syndrome hémolytique dont l'expression clinique est l′anémie fœtale éventuellement compliquée par une anasarque fœto-placentaire ou à l'extrême une mort fœtale in utéro. Nous rapportons l'observation d'un cas d'allo immunisation Rhésus à 34 SA ayant aboutit un hydrops foetalis, l'extraction fœtale par césarienne en vue d'une exsanguino-transfusion a été réalisée, mais le nouveau né est décédé au cours de l'exsanguino-transfusion. Le dépistage des femmes à risque et l'utilisation d'Immunoglobulines anti D ont permis une réduction importante de l′incidence des accidents d'incompatibilité. La mesure du pic systolique de vélocité dans l'artère cérébrale moyenne a bouleversé la surveillance et la prise en charge prénatale des anémies fœtales secondaires à une allo-immunisation Rhésus. Son utilisation dans la surveillance des cas d'allo-immunisation Rhésus permettrait ainsi de réserver les procédures invasives (cordocentèse) comme geste thérapeutique qui permet la transfusion fœtale in utéro. Grâce à une collaboration multidisciplinaire cohérente, l'extraction fœtale peut être programmée, ce qui permet une prise en charge adéquate et rigoureuse, même des nouveaux nés avec atteinte sévère.

## Introduction

L'allo-immunisation fœto-maternelle dans le système RH est la première cause d'anémie fœtale néonatale. Elle est responsable d'un syndrome hémolytique fœtal de gravité variablequi se manifeste in utéro par un état d'anasarque pouvant aller jusqu’à la mort fœtale in utéroet à la naissance d'un ictère hémolytique. La situation la plus classique de l'allloimmunisationfoeto-maternelle concerne l'antigène D[1]. Cependant, des phénomènes d'alloimmunisation peuvent survenir dans d'autres groupes érythrocytaires[2]. Bous rapportons l'observation d'un cas d'alloimmunisation fœto-maternelle dans le système RHayant aboutit à un décès néonatal au cours de l'exsanguino-transfusion dans un contexte d'anasarque fœto-placentaire.

## Patient et observation

Une patiente de 41 ans, G4P3, 1 enfants vivant, de groupe AB Rh-, la première grossesse c'est soldé par un accouchement par voie basse d'un nouveau né vivant mais l'anti D n'a pas été reçu, les deux autres grossesses ont été marquées par un décès néonatal respectivement à j7 et J1 de vie, dans un contexte d'ictère hémolytique.

La quatrième grossesse est la grossesse actuelle, était mal suivie. La patiente était vue en consultation prénatale pour la première fois à 32SA où elle avait bénéficié d'une échographie obstétricale qui a objectivé une anasarque foeto-placentaire et adressée à notre formation pour prise en charge. Elle a consulté dans notre formation à 34 SA. A l'examen clinique la hauteur utérine était excessive à 31cm avec un ballotement fœtal perceptible à la palpation abdominale, les bruits cardiaques fœtaux étaient réguliers mais sourds.

**Figure 1:**
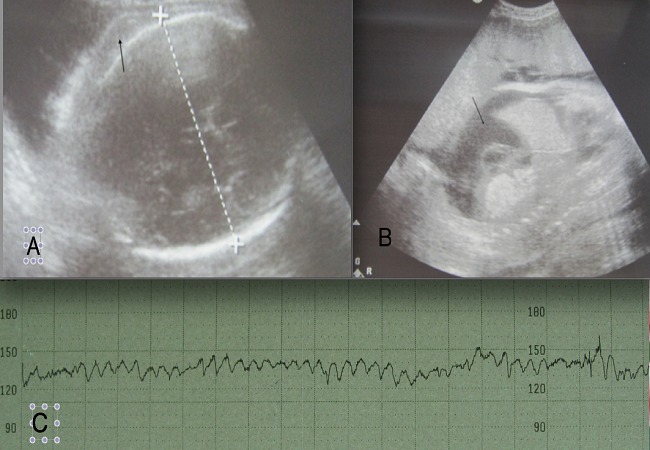
A) Image échographique de la tête fœtale montrant un œdème du scalp (flèche); (B) image échographique montrant l'ascite de grande abondance (flèche) sur une coupe sagittale de l'abdomen; (C) enregistrement du rythme cardiaque fœtal de type sinusoïdal

L’échographie obstétricale a objectivé une grossesse évolutive en présentation de siège. La biométrie fœtale correspondait à l’âge gestationnel pour le diamètre bipariétal (BIP= 87mm) et pour la longueur du fémur (LF = 66mm). Cependant la circonférence abdominale était supérieure au 90ème percentile de 34SA (CA = 423mm). Le fœtus était en état d'anasarque avec présence d’épanchement pleural bilatéral, péricardique et une ascite ainsi qu'un œdème sous cutané diffus (Figure 1 A et B). L'exploration des viscères avait objectivé une hépatomégalie. La veine ombilicale était dilatée mesurant 11mm. Le placenta était épaissi mesurant 80mm. L'hydramnios était manifeste avec un index du liquide amniotique à 350mm. Le doppler ombilical était normal avec un indexe de résistance à 0,64 et le doppler cérébral a montré une élévation du pic systolique de vélocitémesuré sur l'artère cérébrale moyenne arrivant à 80 cm/s. Le rythme cardiaque fœtal était franchement pathologique de type sinusoïdal (figure 1 C). Sur le bilan biologique la recherche d'agglutinines irrégulières était positive, cependant le titrage n'a pu être réalisé.

**Figure 2:**
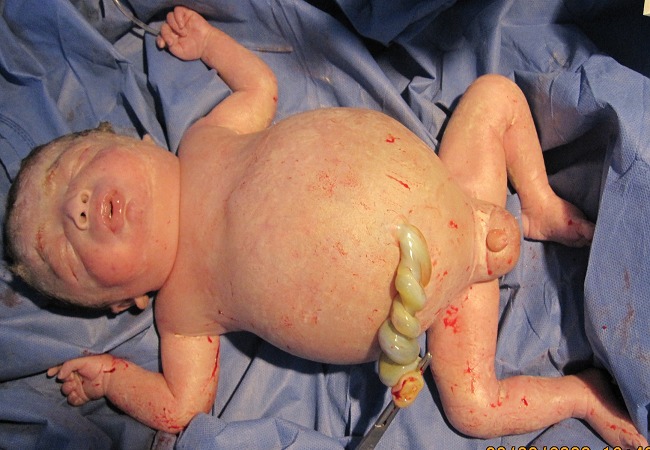
Le nouveau né en hydrops foetalis

La césarienne a été réalisée le jour même, elle a permis l'extraction d'un nouveau-né vivanten hydrops fœtalis (Figure 2), le score Apgarétait à 5/10 à la naissance et à 5min. L'hémogramme et la demande de culots globulaire ont été réalisés su sang du cordon. Le nouveau-né était hypotonique très peu réactif, avec respiration spontanée irrégulière et un rythme cardiaque fœtal à 120 bpm. Il présentait à la naissance des œdèmes étendus et une distension abdominale importante, sans ictère cutané. Il a été intubé et ventilé et transféré en réanimation. En réanimation, un cathéter ombilical a été posé afin de réaliser une exsanguino-transfusion. Il a été réalisé également une ponction pleurale bilatérale. Le taux d'hémoglobine était à 6g/dl. Au moment de commencer l'exsanguino transfusion, le nouveau né a présenté un arrêt cardio-circulatoire non récupéré. Il est décédé à H1 de vie.

## Discussion

### Physiopathologie – Étiologie

*Le système Rhest constitué* de deux gènes contigusRHD et *RHCE* présents sur le chromosome 1. Le gène *RHD* code pour la protéine D (RH1) qui constitue le groupe RhD positif (RH: 1) au sujet qui la possède. Les individus ne possédant pas le gène *RHD* sont doncRhD négatif (RH: -1). Le second gène *RHCE* porte les antigènes C (RH2), E(RH3), c (RH4) et e (RH5) en formant quatre combinaisonsCE, Ce, cE, ce [3].

La séquence des événements qui aboutit à l'alloimmunisation fœto-maternelle se déroule selon les étapes suivantes: 1) passage d'hématies fœtales à travers le placenta; 2) réponse immunitaire primaire maternelle; 3) réponse immunitaire secondaire, habituellement lors d'une grossesse ultérieure, lorsque le fœtus exprime l'antigène RhD produisant des immunoglobuline de type IgG; 4) passage des IgG à travers le placenta et fixation sur les hématies foetales; 5) déclenchement de la maladie hémolytique avec ses conséquencespour le fœtus ou pour le nouveau-né. Les macrophages de la rate et du foie qui vont capter les globules rouges fœtaux fixant les IgG-antiD, entrainant leur hémolyse intra tissulaire et par conséquent une anémie fœtale de gravité variable. Des phénomènes de compensation seront déclenchés pour lutter contre l'anémie fœtale; L’érythropoïèse accru dans les tissus hématopoïètique (Foie, rate) vont entrainer un dysfonctionnement hépatique (hypoalbuminémie) et une hypertension portale également de gravité variable aboutissant au tableau classique d'hydrops foetalis avec épanchement des séreuses, œdème cutané diffus, hépato-splénomégalie, hydramnios et épaississement placentaire [3]. A la naissance, l'immaturité du foie fœtal limite la conjugaison de la bilirubine produite lors de l'hémolyse. Son accumulation dans la circulation fœtale entraine un ictère néonatal secondaire responsable d'ictère nucléaire.

Cette succession d’événements concerne donc au moins deux contacts antigéniques dans cette forme habituellede la maladie. Le premier contact s'effectue au cours de la première grossesse ou de l'accouchement; car il existe un passage physiologique de quelques globules rouges fœtaux dans la circulation maternelle. Ce passage est augmenté parallèlement avec l’âge de la grossesse [3], lors des interventions ou traumatisme sur utérus gravide et lors des métrorragies. En dehors de la grossesse, les transfusions de produits sanguins sont devenues actuellement des situations exceptionnelles d'allo immunisation.

### Le dépistage des grossesses à risque d'alloimmunisation

Commence par la détermination du groupe sanguin de la mère en début de grossesse, ainsi que celui du père en cas de mère Rh:-1. Il est actuellement possible de réaliser une détermination du groupe sanguin fœtal dans le sang maternel dès la 10ème SA par PCR.

La recherche des agglutinines irrégulières (RAI) est la technique de choix pour la surveillance des grossesses à risque. En effet, l'absence d'agglutinine irrégulière élimine toute atteinte fœtale. Toutes les femmes enceintes doivent bénéficier d'une recherche des agglutinines irrégulière en début de grossesse. Pour les femmes de groupe Rh:-1, cet examen doit être répété chaque mois [2]. La positivisation des RAI au cours de la grossesse témoigne de la survenue d'alloimmunisation dont il faut apprécier la gravité.

La titration des agglutinine irrégulière est réalisée dès que la recherche des agglutinine irrégulière est positive. Au delà d'un titre de 8, Il est préférable de réaliser un dosage pondéral des anticorps. Cependant ce dosage n'est valable que pour les immunisations dans le système Rh:1. Il existe une certaine corrélation entre ces deux examens et le degré de gravité de l'immunisation [4]. En effet, le risque d'atteinte fœtal est minime si le titre est inférieur à 16 et si le dosage pondéral est inférieur à 1µg/ml. Un dosage pondéral supérieur à 5µg/ml témoigne d'une atteinte fœtale sévère [3]. Le rythme de surveillance biologique des grossesses conflictuelles dépend de la sévérité de l'atteinte; si le titre reste inférieur à 16, une titration est réalisée chaque mois puis chaque deux semaine à la deuxième moitié de la grossesse [4].

### L’évaluation de la sévérité de l'atteinte fœtale

L'appréciation du degré de l'anémie fœtal a été révolutionnée par la mesure du pic systolique de vélocité de l'artère cérébrale moyenne (ACM) au doppler [5]. Contrairement aux méthodes invasives; amniocentèse et cordocentèse, La mesure du pic systolique de vélocité de l'ACM et une technique non invasive reproductible, ayant une sensibilité de 91% et une spécificité de 100% dans la détection des anémies fœtales sévères [6]. L'augmentation du pic systolique de L'ACM serait due à l'augmentation du débit cardiaque et à la diminution de la viscosité sanguine par diminution de l'hématocrite fœtale [6, 7]. En effet, le risque d'anémie fœtale sévère est accru si le pic systolique de vélocité de l'ACM est supérieur au 1,5 multiple de la médiane (MoM) pour le même âge gestationnel projetée sur la courbe de Mari. Le rythme de surveillance par le doppler est variable en fonction de la sévérité de l'atteinte. Une surveillance bimensuelle au delà de 20 SA si l'immunisation est modérée. La surveillance est hebdomadaire si le risque d'anémie fœtale est plus important avec dosage bimensuel des anticorps [8].

L’échographie fœtale recherche les signes d'intolérance fœtale à l'anémie; l‘hydramnios, l'anasarquefœto-placentaire et l'hépato-splénomégalie qui sont des signes tardifs prédicteur d'anémie fœtale sévère, contrairement à la mesure de l’épaisseur du placenta et de la veine ombilicale [7].

L’étude du rythme cardiaque fœtal permet la surveillance du bien être fœtal dès que les signes d'anémies fœtale. Les anomalies sont variables, et réalisent à l'extrême un rythme sinusoïdal.

Les procédés invasifs intraovulairessont actuellement réservés à une visée thérapeutique.

*La prise en charge thérapeutique* doit débuter en anténatal, la décision de transfusion in utéro est envisageable dès 17 SA-20SA jusqu′à 34 SA [3]. Il s'agit d'un geste invasif qui consisté à ponctionner in utéro la veine coronale et à transfuser des globules rouges irradiés de groupe O et de phénotype identique à celui maternel. Avant la transfusion, l'anémie doit être confirmée par un dosage direct sur le sang fœtal. Ce procédé n'est pas dénué de risque, de mort fœtale in utéro ou d'accouchement prématuré évalué à 3% [8]. Il peut s'agir d'une transfusion in utéro simple (TIU) ou avec soustraction du sang fœtal. L'exsanguino-transfusion in utéro (ETIU) à l'avantage de limiter la surcharge volémique fœtale mais elle expose à un risque fœtal plus important limitant son utilisation [8]. L'extraction fœtale,en cas d'atteinte sévère, est programmée dès que le risque de grande prématurité est écarté. Pour les attentes modérées et légère il n'y a aucun intérêt à maintenir la grossesse au delà du terme de 37 SA, car le risque d'aggravation brutale existe toujours.

La prise en charge postnatale est axée sur deux principaux paramètres; la correction de l'anémie fœtale et del'hyperbilirubinémie. La réalisation de photothérapie précoce et intensive permet de réduire le taux de bilirubine fœtale. La transfusion fœtale est indiquéeencas d'anémie fœtale mal tolérée. L'exsanguino transfusion est réservée aux cas d'anasarque avec anémie profonde et d'hyperbilirubinémie menaçante d'ictère nucléaire résistante au traitement par photothérapie [9]. Le recours à l'exsanguino-transfusion a régressé depuis l’élargissement des transfusions fœtales in utéro.

### La prophylaxie

L'application stricte des mesures de prophylaxie par injection d'immunoglobuline anti-D a permis de réduire l'incidence de l'alloimmunisation fœto-maternelle. Elle répond à des standards régulièrement actualisés. Le collège national des gynécologues et obstétriciens français recommande depuis 2005 l'utilisation d'immunoglobuline anti D chez les patientes Rhésus négatif chez qui la recherche d'agglutinines irrégulières est négative selon un protocole bien établi. Au cours de la grossesse, l'injection de 200µg d'immunoglobuline anti D n'est réalisée qu'après un incident favorisant le passage de globules rouges fœtales à la circulation maternelle au premier et au deuxième trimestre. Au troisième trimestre de la grossesse l'injection de 300µg d'immunoglobuline anti-D est systématique à 28 SA. Après l'accouchement une injection de 200µg d'immunoglobuline anti-D est administrée systématiquement après la naissance, une dose supplémentaire sera administrée ultérieurement en fonction du résultat du test de Kleihauer et après vérification du groupe sanguin fœtal [3].

## Conclusion

L'allo-immunisation fœto-maternelle anti-érythrocytaire reste d'actualité du fait de leur persistance malgré les mesures prophylactiques et du fait des progrès considérables dans les méthodes de prise en charge.
